# A novel method for screening beta-glucosidase inhibitors

**DOI:** 10.1186/1471-2180-13-55

**Published:** 2013-03-08

**Authors:** Sony Pandey, Ayinampudi Sree, Soumya S Dash, Dipti P Sethi

**Affiliations:** 1 Environment and Sustainability Department, CSIR-Institute of Minerals and Materials Technology, Bhubaneswar 751013, India

**Keywords:** Glucosidase inhibitor, Esculin, Conduritol β-epoxide, Microbial extracts, marine microorganisms, anti-diabetics, anti-obesity, anti-HIV

## Abstract

**Background:**

Few beta-glucosidase inhibitors have so far been reported from microorganisms due to the practical difficulties in performing the inhibition tests and subsequent interpretation of results. In an effort to investigate marine microbial extracts for β-glucosidase inhibitors, we developed a new protocol, using esculin as substrate in an agar plate based assay, to screen a large number of microbial extracts in a short span of time.

**Results:**

With the new method, pale yellowish zones against the blackish brown background could be visually observed with more clarity in sample extracts where β-glucosidase inhibitor was present. The new method was compared with the closest existing method and established beyond doubt. This agar plate based procedure required about one hour for minimum 12 samples and the throughput increases with the size of the agar gel plate used.

**Conclusions:**

The new protocol was simple, rapid and effective in detecting beta-glucosidase inhibitors in microbial extracts.

## Background

Glucosidase inhibitors are responsible for disruption of the activity of glucosidase, an enzyme that cleaves the glycosidic bond. These inhibitors have played a vital role in revealing the functions of glucosidases in living system by modifying or blocking specific metabolic processes; and, this revelation led to several applications of these chemical entities in agriculture and medicine [1].

The quest for new glucosidase inhibitors is crucially important owing to their therapeutic potential in the treatment of diabetes, human immuno deficiency virus infection, metastatic cancer, lysosomal storage disease etc. [2]. Microorganisms, particularly marine microorganisms, have an unparalleled distinction of producing valuable compounds. So, screening microbial culture extracts for uncovering novel structures that can inhibit glucosidases, is of immense interest.

There are extremely few reports of glucosidase inhibitors, particularly β-glucosidase inhibitors from microorganisms, possibly because of lack of efficient high throughput methods to detect the presence of β-glucosidase inhibitors in microbial culture extracts. The most commonly employed method involves *p*-nitrophenyl-*β*-D-glucopyranoside (PNPG) as substrate in either microplate screening test or TLC autographic method [3-5]. In this method, glucosidase activity is measured indirectly, in a colorimetric assay by visual or spectrophotometric assessment of the nitrophenyl chromophore (yellow) released from PNPG in the absence of inhibitor. The yellow colouration developed using this glucopyranoside in a glucosidase positive reaction, is too faint and not in contrast with its surrounding for clear visual distinction in TLC plate or otherwise [5-7]. Microwell plate methods are rapid, but many factors such as protease in fermentation broths, microbial contamination of extracts, biological pigments, or salts in crude extracts can interfere with the readings [8].

The TLC autographic method - using esculin as substrate - by Salazar and Furlan [7] was the most convincing method as an alternative to the methods using PNPG. In this TLC autographic method, the enzyme β-glucosidase is immobilized by gel entrapment in agar and TLC autography is performed. The enzyme activity is tested on esculin (6, 7-dihydroxycoumarin 6-glucoside) as substrate which splits into esculetin (6, 7-dihydroxycoumarin) and glucose; the released esculetin reacts with FeCl_3_ to form a blackish brown precipitate. Inhibition of this activity is observed as a pale yellowish zone around the spot of the positive samples.

Many of the previous studies have used TLC autographic method, which may not be suitable for high throughput screening as they are more laborious and time consuming. Moreover, uniform separation of compounds in all extracts cannot be achieved with single solvent system; hence spotting all the extracts on one TLC plate to rapidly perform the assay would be frustrating. For screening a large number of natural extracts, TLC autography was performed without developing the plate so that activities resulting from synergistic action of multiple components of extracts are detected [9]. In this context, we consider the use of TLC plate to be unnecessary; more so because the zone of inhibition on white TLC plate background was not very clear and hence there are chances of losing some promising natural extracts. In a nutshell, accurate assessment of glucosidase inhibition activity in several extracts at a time is difficult by these conventional methods.

Thus, we developed a novel method by pouring the enzyme-agar solution in a thin layer on a petri dish and spot inoculating the samples on the agar surface, for achieving clear detection of β-glucosidase inhibitors in microbial culture extracts.

## Results and discussion

**Figure 1:**
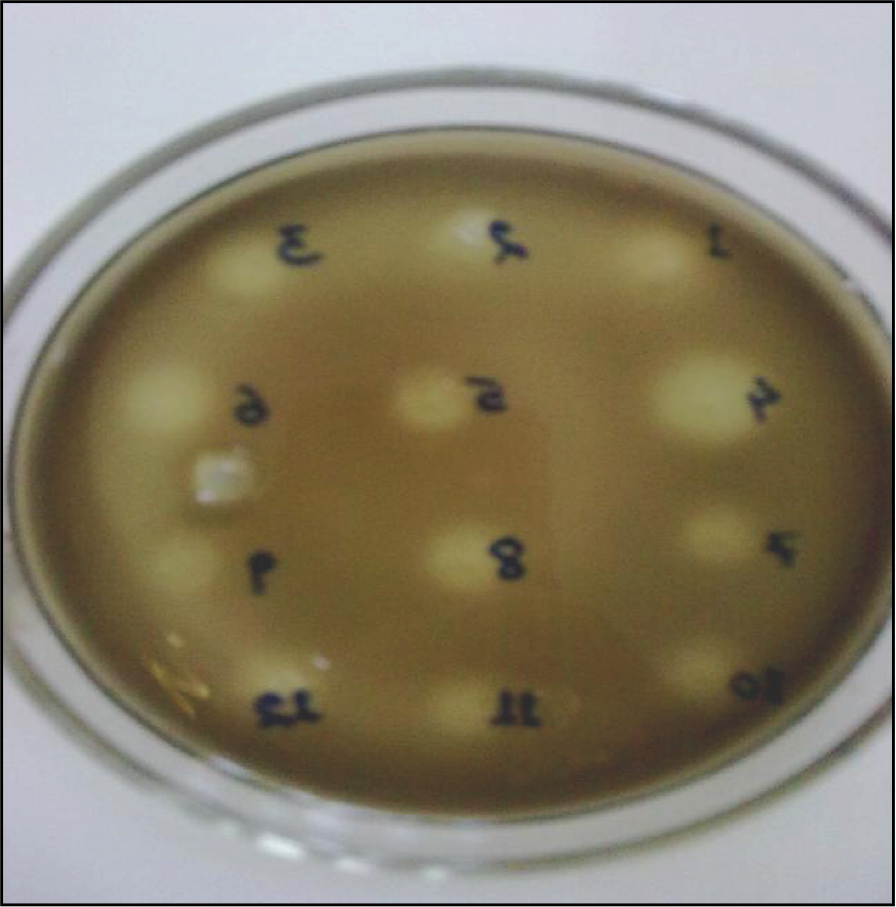
β-glucosidase inhibition using the agar plate method developed in this study.

The microbial culture extracts, which were positive for β-glucosidase inhibitors, showed as pale yellowish zone of inhibition at places where the samples were spotted while the rest of the plate turned blackish brown due to the reaction of esculetin and ferric ion (Figure 1). A large number of methanol extracts of microorganisms were screened using the new method, and we found 98 extracts (32%) contain inhibitors out of 304 extracts tested (data not shown). As compared to the earlier reports of screening plant and microbial extracts, this method could detect greater number of positive extracts, which may be, because of the easily discernible results [3,8]. This method is also rapid as it takes about 1 hr to test 12 samples in a Ø90 mm petri plate. The throughput can be increased by increasing petri plate size or using a multiple of plates.

**Figure 2:**
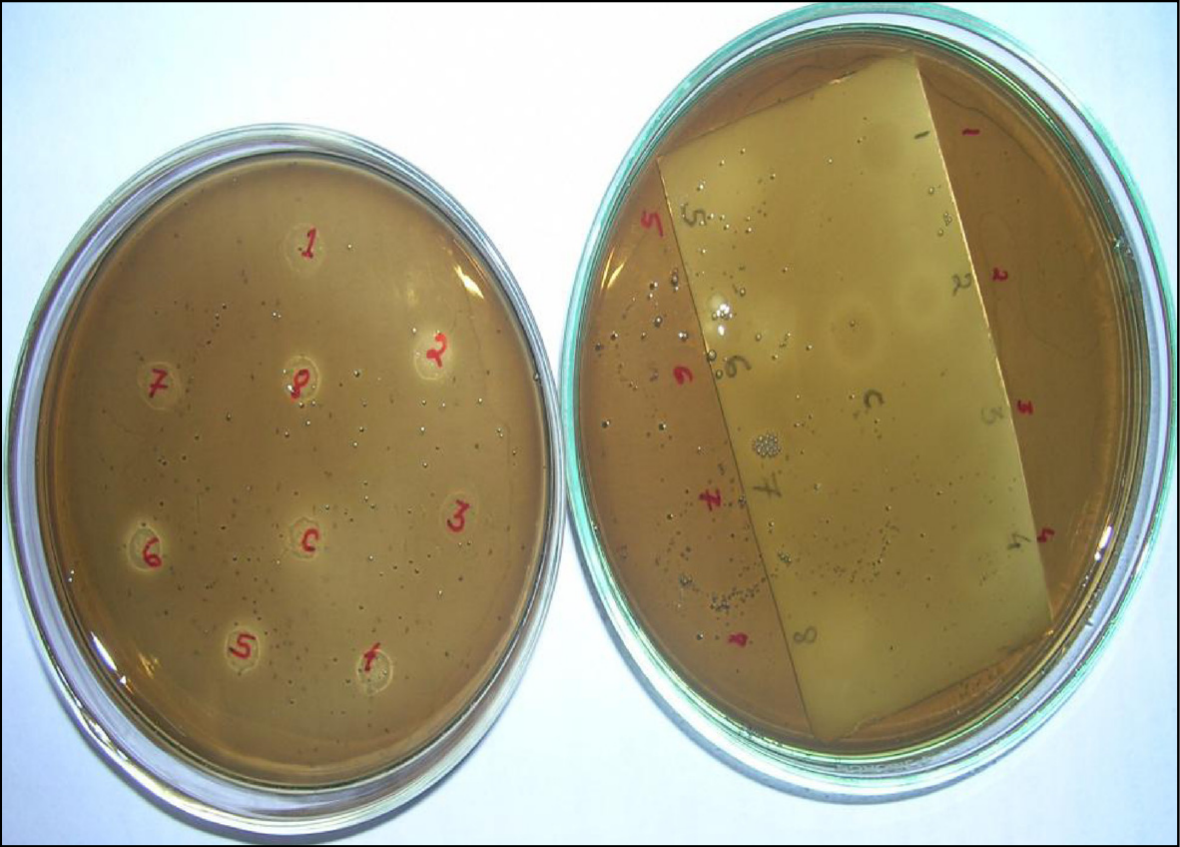
**Side by side comparison of agar plate method with TLC autography method.** Samples labelled as 1, 2, 3, 4, 5, 6, 7 and 8 are the methanol extracts of marine microorganisms and, C is for control - 0.75 μg conduritol β-epoxide.

**Figure 3:**
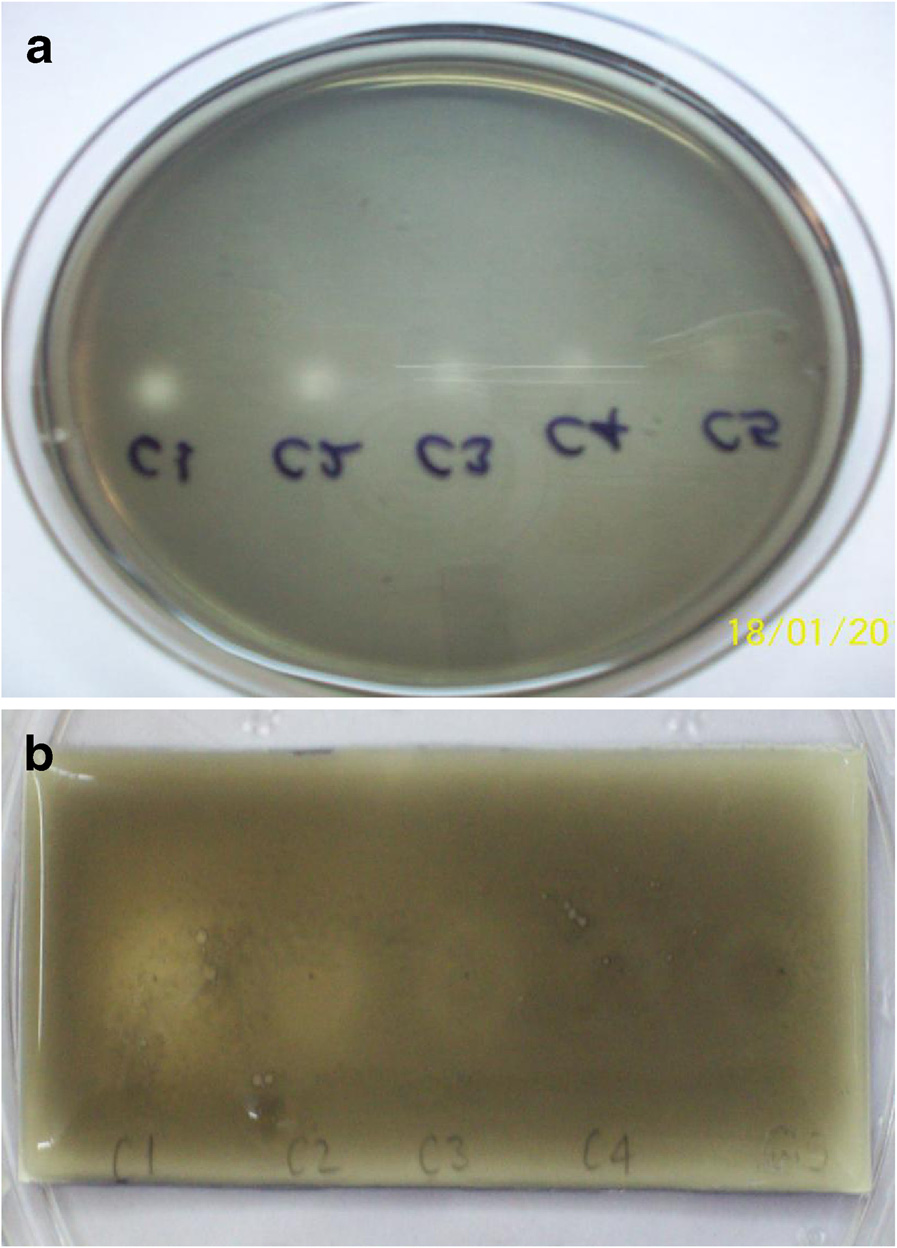
**Conduritol β-epoxide in different doses in: a) agar plate method - samples spot inoculated on the agar surface b) TLC autography method.** C1 - 2.5 μg, C2 - 1.0 μg, C3 - 0.50 μg, C4 - 0.10 μg and C5 – 0.05 μg.

The present agar plate based method evolved from the protocol described by Salazar and Furlan [7], since we encountered some difficulty while screening the microbial extracts. The enzyme-agar solution did not evenly spread on the TLC plate, and the brown colour (due to esculetin reaction) on white plate background was not uniform throughout the TLC plate; thus it was difficult to observe the inhibition activity as clear spots in contrast to the surrounding. Although zones were visible, it was difficult to ascertain certain samples as positive or negative. Hence we modified the method, and used petri plates to set in the enzyme-agar solution and spot inoculated the samples on the enzyme-agar plate and dried the samples using a blow-dryer. Then the plate was flooded with substrate solution. The results were visually clear in this agar plate method when compared side by side with TLC autography (see Figure 2 and Figure 3).

We tested a subset of 31 samples with Salazar’s method described in 2007 and 2011 [7,9], as well as with the new method. All of the 31 samples were inactive when the TLC plate was developed indicating synergistic interaction among the sample components was responsible for the positive activity. Out of the 31 extracts tested 13 were observed to be positive on the undeveloped TLC plate whereas, 16 showed β-glucosidase inhibition activity on the agar plate method. However, the quality of zone in some samples was not clear in TLC autographic method as shown in Figure 2.

Conduritol β-epoxide - an active site-directed covalent inhibitor - was tested in a dose dependent order to confirm the effectiveness of this method and the results are presented (Table 1). The minimum detection limit of conduritol β-epoxide in the new method, when samples were spot inoculated on the agar surface, is 0.05 μg. Although Salazar and Furlan [7] reported the detection limit to be 0.10 μg in TLC autographic method, we observed similar results with conduritol in both the methods. However, the clarity of zones is undoubtedly better in the agar plate method as seen in Figure 3a and 3b.

**Table 1:** Inhibition of β-glucosidase by different doses of conduritol β -epoxide

	**Concentration (μg)**						
	**2.5**	**1**	**0.75**	**0.50**	**0.25**	**0.1**	**0.05**
Inhibition	+	+	+	+	+	+	+

**Figure 4:**
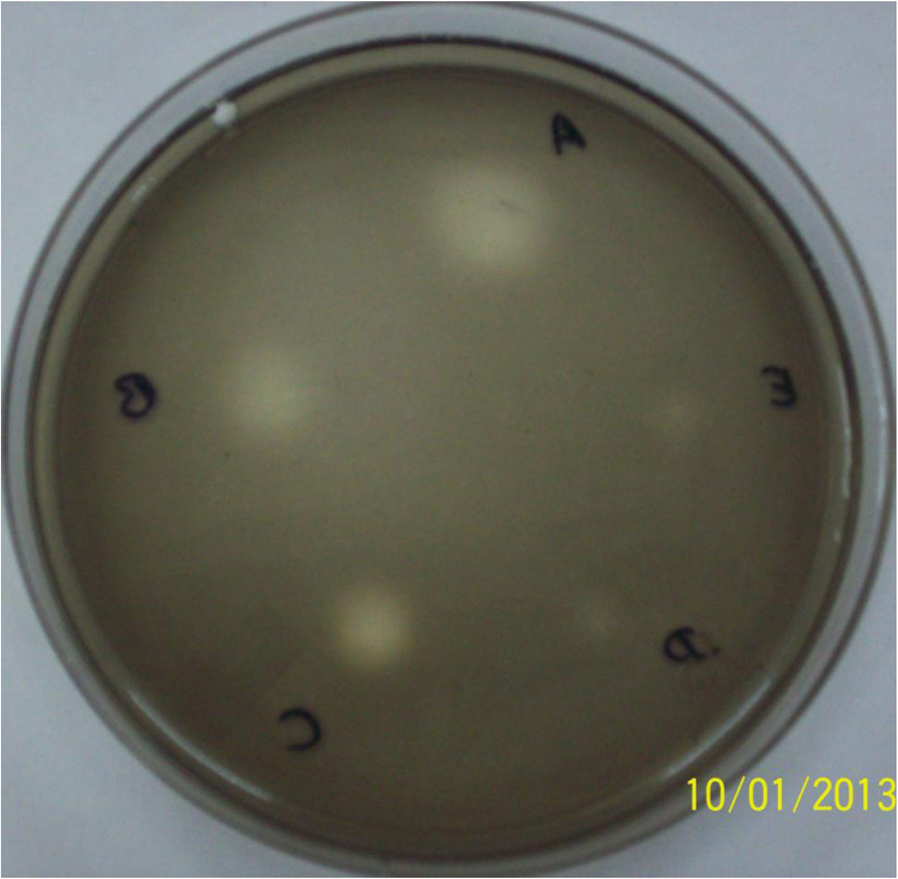
**1-(3-aminopropyl)-imidazole in different doses. A -** 2000 μg, **B -** 1000 μg, **C -** 500 μg, **D -** 100 μg and **E-** 50 μg.

We also tested imidazole derivatives, 1-(3-aminopropyl)-imidazole and 2-aminobenzimidazole, as reversible inhibitors of β-glucosidase with this method [10]. Figure 4 demonstrates the inhibition activity of 1-(3-aminopropyl)-imidazole in a dose dependent order up to 50 μg. The detection limit of 2-aminobenzimidazole was 100 μg. As compared to conduritol, imidazole derivatives are less potent inhibitors of β-glucosidase [11].

Comparing the new method with the protocol of Salazar and Furlan [7], we achieved reliable results in lesser time. The enzyme-inhibitor and enzyme-substrate reaction time of 2 hrs was not necessary. The enzyme-inhibitor incubation of 15 min was sufficient as the samples were blow dried. Similarly, after pouring the esculin solution the zones could be seen within 10–15 min, which off course becomes clear as the time progresses, but within 30 min, the contrast of zones is completely clear.

## Conclusions

The new method can be used in conjunction with TLC autography. With agar plate method, several extracts could be quickly screened for activity and then the compound responsible for β-glucosidase inhibition in positive extracts could be located with the TLC autographic method. The present method is rapid and effective; hence it is suitable for initial screening. The contrast in inhibition zones is quite prominent as compared to other methods described so far for β-glucosidase inhibition. The sensitivity of this method is same or better than the TLC autographic method. It is very simple and convenient to perform.

## Methods

### Materials

Almond β-glucosidase enzyme (5.2 U/mg, Sigma) reconstituted in sodium acetate buffer to 2.5 U/ml, 0.1 M sodium acetate buffer (pH-5), 0.2% w/v solution of esculin (HiMedia, Mumbai), 0.5% w/v solution of FeCl_3_, conduritol β-epoxide (Sigma) in 5 mg/ml solution and agar powder.

### Revival of cultures

A total of 304 marine microorganisms isolated from two sponge samples and 4 sediment samples were revived from cryopreserved stocks (in 10% glycerol) and agar slants. All the organisms grew on Nutrient Agar (HiMedia) media prepared in 50% aged natural seawater at 30°C within 48–72 hrs.

### Extraction of metabolites

The organisms were inoculated in 50 ml Nutrient Broth prepared in 50% natural seawater and incubated at 30°C in 200 rpm shaker for 48 hrs. The culture was centrifuged (9000 rpm/20 min) and the supernatant used for extraction of secondary metabolites. The supernatant collected in a 250 ml flask was extracted by mixing 10% diaion HP-20 (Sigma) and shaking for 30 min on a magnetic stirrer. Then the flask contents were packed in a glass column and washed with 15 ml distilled water. Finally, the metabolites on diaion were eluted with 20 ml methanol. The collected methanol fractions were evaporated in a rotary evaporator (Heidolph, Germany), dissolved in DMSO and stored at - 20°C.

### Inhibition assay

An enzyme agar solution containing β-glucosidase was prepared in 7 ml sodium acetate buffer with 0.07 g of agar powder dissolved at 80-100°C; followed by the addition of 1.2 ml of FeCl_3_ solution and 40 μl of enzyme β-glucosidase at 60°C (0.01 U/ml). The final volume was adjusted to 10 ml with the acetate buffer. An aliquot of 8–10 ml solution was poured into petri plates and allowed to set. The samples - 5 μl of the extract - were spot inoculated with a micropipette on the surface of the agar plate and blow dried or air dried. Alternately, the samples can be loaded on sterile filter paper discs, dried and placed on the agar plate. The plates were incubated at room temperature for 15 min for primary reaction between the enzyme and inhibitor. Later on, 6–7 ml of esculin solution was added to cover the surface of agar and again incubated at room temperature for 30 min for enzyme-substrate reaction. In case, paper discs are used they have to be removed before adding esculin. Conduritol β-epoxide, an irreversible inhibitor, in concentrations 2.5, 1, 0.75, 0.50, 0.25, 0.10 and 0.05 μg was used as a positive control and DMSO without extract as negative control. 1-(3-aminopropyl)-imidazole and 2-aminobenzimidazole were used as reversible inhibitor control, in concentrations 2000, 1000, 500, 100 and 50 μg. Clear zones of inhibition were recorded by measuring the zone size. A subset of 31 samples was also compared using this agar plate method and TLC autographic method with or without developing the TLC plate. These experiments were repeated thrice with some extracts to check the reproducibility of the method.

## Competing interests

The authors declare no competing interests.

## Authors’ contributions

SP contributed to the design of experiments, acquisition, analysis and interpretation of data, and drafting the manuscript. AS contributed in the conception of work on beta-glucosidases, sample collection and editing of the manuscript. SSD and DPS helped in execution of experimental work and acquisition of data. All authors have read and approved the final manuscript.
